# Effects of vitamin C and E supplementation on endogenous antioxidant systems and heat shock proteins in response to endurance training

**DOI:** 10.14814/phy2.12142

**Published:** 2014-10-07

**Authors:** Kristoffer T. Cumming, Truls Raastad, Geir Holden, Nasser E. Bastani, Damaris Schneeberger, Maria Paola Paronetto, Neri Mercatelli, Hege N. Østgaard, Ingrid Ugelstad, Daniela Caporossi, Rune Blomhoff, Gøran Paulsen

**Affiliations:** 1 Department of Physical Performance, Norwegian School of Sport Sciences, Oslo, Norway; 2 Department of Nutrition, Institute of Basic Medical Sciences, University of Oslo, Oslo, Norway; 3 Department of Movement, Human and Health Sciences, University of Rome “Foro Italico”, Rome, Italy; 4 Division of Cancer Medicine, Surgery and Transplantation, Oslo University Hospital, Oslo, Norway; 5 Norwegian Olympic Sports Center, Oslo, Norway

**Keywords:** Antioxidant enzymes, gene expression, NF*κ*B, stress proteins

## Abstract

Reactive oxygen and nitrogen species are important signal molecules for adaptations to training. Due to the antioxidant properties of vitamin C and E, supplementation has been shown to blunt adaptations to endurance training. In this study, we investigated the effects of vitamin C and E supplementation and endurance training on adaptations in endogenous antioxidants and heat shock proteins (HSP). Thirty seven males and females were randomly assigned to receive Vitamin C and E (C + E; C: 1000 mg, E: 235 mg daily) or placebo (PLA), and underwent endurance training for 11 weeks. After 5 weeks, a subgroup conducted a high intensity interval session to investigate acute stress responses. Muscle and blood samples were obtained to investigate changes in proteins and mRNA related to the antioxidant and HSP system. The acute response to the interval session revealed no effects of C + E supplementation on NF*κ*B activation. However, higher stress responses to exercise in C + E group was indicated by larger translocation of HSPs and a more pronounced gene expression compared to PLA. Eleven weeks of endurance training decreased muscle GPx1, HSP27 and *α*B‐crystallin, while mnSOD, HSP70 and GSH remained unchanged, with no influence of supplementation. Plasma GSH increased in both groups, while uric acid decreased in the C + E group only. Our results showed that C + E did not affect long‐term training adaptations in the antioxidant‐ and HSP systems. However, the greater stress responses to exercise in the C + E group might indicate that long‐term adaptations occurs through different mechanisms in the two groups.

## Abstract (precis)

Reactive oxygen species are important signal molecules for adaptations to training. Previously vitamin C and E supplements has been shown to blunt adaptations to endurance training. In this study, we investigated the effects of vitamin C and E supplementation and endurance training on adaptations in endogenous antioxidants and heat shock proteins.

## Introduction

Metabolic demands during exercise are associated with augmented formation of reactive oxygen and nitrogen species (RONS; Davies et al. 1982). High levels of RONS may cause oxidative damage to proteins, nucleotides, and lipids; and ultimately RONS can impair cell functions or induce necrosis (Peternelj and Coombes 2011). To counteract these apparently negative effects of high intensity exercise, it is widely common among athletes to use dietary vitamin/antioxidant supplements (Sobal and Marquart 1994). However, although increased RONS production during exercise has potential negative effects, transient increases in RONS seem to be a trigger for many exercise‐induced adaptations in skeletal muscle (Powers et al. 2010). Indeed, recent studies indicate that antioxidant supplementation may blunt mitochondrial biogenesis induced by endurance training in both animal and human models (Gomez‐Cabrera et al. 2008; Paulsen et al. 2014).

Improved aerobic capacity appear to occur concomitantly with up‐regulation of endogenous antioxidant systems (Powers and Jackson 2008) and heat shock proteins (HSP) in skeletal muscle (Morton et al. 2009b). While the main role of the antioxidant enzymes is to decrease oxidation and prevent oxidative damage, HSPs can prevent and reverse damage to proteins. Intriguingly, the HSPs cooperate with the antioxidant systems, and collectively they have essential roles in cell homeostasis. Up‐regulation of these proteins is, therefore, important adaptations for increased protection and recovery capacity in face of cellular stress and damage induced by high‐intensity exercise (Powers and Jackson 2008; Morton et al. 2009b).

Like in mitochondrial biogenesis, alterations in redox status modulate the training‐induced adaptations in antioxidant systems and HSP systems (Gomez‐Cabrera et al. 2005; Fittipaldi et al. 2014). A potential target for antioxidant supplementation might be the stress‐ and RONS sensitive NF*κ*B p65 pathway (nuclear factor kappa B p65/RelA), which can activate gene expression of the antioxidant enzymes manganese superoxide dismutase (mnSOD) and glutathione peroxidase 1 (GPx1; Wan et al. 1994; Zhou et al. 2001) and HSP70 (Tranter et al. 2010; Sasi et al. 2014). Furthermore, HSP gene expression seems to also be activated via HSF1 with increased RONS levels (Jacquier‐Sarlin and Polla 1996). This indicates that antioxidants have the potential to modulate the expression of stress‐related proteins by decreasing RONS levels.

In agreement with this assumption, antioxidant supplements appear to inhibit adaptations in both antioxidant enzymes and the heat shock proteins (Peternelj and Coombes 2011), although the literature is equivocal. Some authors claim that antioxidant supplementation blunt resting mRNA expression of *SOD2* (mnSOD), *GPx1* and *HSPA4* (HSP70; Gomez‐Cabrera et al. 2005, 2008; Fischer et al. 2006; Ristow et al. 2009), while others document no interference on protein levels (Jackson et al. 2004; Fischer et al. 2006; Yfanti et al. 2011, 2012; Gliemann et al. 2013). Furthermore, antioxidants have been shown to abolish the acute exercise‐induced increases in HSP60 and HSP70 protein levels (Khassaf et al. 2003; Jackson et al. 2004).

In addition to the intra‐cellular systems in muscle fibers, the plasma offers a variety of different antioxidant systems, like glutathione (GSH) and uric acid. Intriguingly, vitamin C supplementation effectively decrease uric acid levels (Juraschek et al. 2011). Research on the combined effects of antioxidant supplements and endurance training on GSH and uric acid levels in healthy individuals is, however, lacking, and further studies are needed to shed light over a possible interaction effect.

Due to the conflicting data and variations in methods used in previous studies, further investigation is needed to understand how antioxidant supplementation potentially affects both the acute stress response and the training adaptations in antioxidant systems and the heat shock proteins. This knowledge could have both clinical and sport‐related interest, as these proteins are important to prevent damage and optimal recovery processes.

Consequently, the aim of this study was to investigate the effects of vitamin C and E supplementation on adaptations in the endogenous antioxidant systems and heat shock proteins after 11 weeks of endurance training. Further, we wanted to investigate how the supplements would affect acute stress responses to a high‐intensity interval session midway into the training intervention. We hypothesized that vitamin C and E supplementation would decrease the exercise‐induced increase of RONS, and thereby blunt the acute NF*κ*B activation and the concomitant increase in gene expression of endogenous antioxidants (*SOD2*,* GPx1*) and heat shock proteins (*HSPA4* and *CRYAB*) in muscle (*m. vastus lateralis*) after a session of high‐intensity interval running. Due to the accumulation of stress over time, muscle heat shock protein (*α*B‐crystallin, HSP27 and HSP70) levels were expected to increase in both groups, but to a lesser degree with vitamin C and E supplementation. Furthermore, we hypothesized that vitamin C and E supplementation over 11 weeks of training would blunt the training‐induced upregulation of endogenous antioxidant systems (mnSOD, GPx1 and GSH) in the muscle and plasma (GSH, uric acid, and total antioxidant capacity).

## Methods

### Participants

Nineteen male and 18 female participants completed the study. All participants were physical active, conducting regularly endurance training (1–4 times per week) before starting the study. Physical activity prior to the study was reported with a questionnaire by the participants. All participants gave written informed consent before entering the study, and were informed about potential risks related to the experiment. The study was approved by the Regional Ethics Committee of Southern Norway and was performed in accordance with the Helsinki Declaration.

### Supplements

The vitamin C and E and placebo pills were produced under Good Manufacturing Practice (GMP) requirements at Petefa AB (Västra Frölunda, Sweden). Each vitamin pill contained 250 mg of ascorbic acid and 58.5 mg DL‐*α*‐tocopherol acetate. The placebo pills had the same shape and appearance as the vitamin pills. All supplements were stored in unlabeled boxes.

The participants ingested two pills (500 mg of vitamin C and 117 mg vitamin E) 1–3 h before every training session and two pills in the hour after training. On nontraining days, the participants ingested two pills in the morning and two pills in the evening. The intake of pills was confirmed with an online training diary. Thus, daily dosage was 1000 mg of vitamin C and 235 mg vitamin E. Compliance to the supplements were 97 ± 5% (Paulsen et al. 2014). The total supplemental dosage of vitamin C was ~13 times higher than the recommended daily dietary allowance in the Nordic countries, and ~23 times higher for vitamin E.

Besides the supplementation given in the study, the participants were informed not to take any form of nutritional supplements. They were told not to drink more than two glasses of juice and four cups of coffee or tea per day. Juices especially rich in antioxidants, such as grape juice, were to be avoided.

### Training

The training protocol used in the present study has been described in detail previously (Paulsen et al. 2014). In short, after a baseline VO_2max_ test, the participants were randomly assigned to receive vitamin C and E or a placebo supplement. The training consisted of three blocks over 11 weeks, where three running sessions were conducted for the first three weeks. Thereafter, the participants increased to four sessions per week. The exercise durations increased linearly in each exercise block. The training sessions were a mix of low‐ (72–82% of HR_max_, 60 min), moderate‐ (82–87% of HR_max_, 30 min), and high intensity (>90% of HR_max_, intervals 4–6 × 4–6 min). Exercise intensity was calculated from maximal heart rate and the participants' perceived exertion using the Borg Scale (RPE). During each training session, heart rate and exercise intensity was monitored and logged using a heart frequency monitor (RS800CX; Polar Electro Oy, Tempere, Finland), and type of exercise and session RPE were logged using an online training log after each training session. For variation and motivation, participants were allowed to do alternative exercise forms (e.g., cycling, cross‐country skiing) once per week, including a maximum of two bouts of other activities in addition to the planned training sessions.

### Acute exercise bout

After 5 weeks of training, a subsample of participants (*n *=**16; five males, 11 females; age 23 ± 2 years, height 172 ± 8 cm, body weight 65 ± 10 kg VO_2max_ 59 ± 8 mL/kg/min) volunteered to complete an exercise bout of 4 × 4 min high‐intensity interval training (>90% of maximal heart rate [HR_max_]). The exercise session was performed on a treadmill (ELG 90/200 Sport; Woodway GmbH, Weil am Rhein, Germany). Exercise intensity was monitored using a heart rate monitor. VO_2_ was measured during the last 1.5 min of each 4‐min interval using a mixing chamber (Oxycon Pro; Erich Jaeger GmbH, Hoechberg, Germany), and blood lactate was measured (YSI 150 Sport Lactate Analyzer; YSI Inc., Yellow Springs, OH) immediately after each interval using a finger stick. The participants ingested the supplements 1–3 h before and immediately after the exercise bout. Blood samples were collected before exercise and 10, 90, and 180 min postexercise. Muscle biopsies were collected pre‐exercise and 90 min postexercise.

### Muscle tissue sampling and handling

Muscle biopsies from the mid‐portion of the right *m. vastus lateralis* were collected under local anesthesia (Xylocain adrenalin, 10 mg/mL + 5 *μ*g/mL; AstraZeneca PLC, London, UK) before and after the training intervention as previously described (Cumming et al. 2014). The posttraining insertion was proximally located to the pre‐training site (approximately 3 cm). For the participants that also took part in the mid‐way, acute exercise bout, biopsies were collected from the left thigh. Tissue intended for mRNA analyses were placed in RNAlater (Cat#AM7020, Ambion; Life technologies, Carlsbad, CA). All muscle samples were stored at −80°C for later analyses.

#### Protein immunoblot and ELISA

Muscle tissue was homogenized and fractionated into cytosol‐, membrane‐, nuclear‐ and cytoskeletal fractions, using a commercial fractionation kit according to the manufacturer's procedures (ProteoExtract Subcellular Proteome Extraction Kit, Cat#539790, Calbiochem; EMD Biosciences, Schwalbach, Germany). Protein concentrations were assessed with a commercial kit (Bio‐Rad DC protein micro‐plate assay, Cat#0113, Cat#0114, Cat#0115; Bio‐Rad, Hercules, CA), a filter photometer (Expert 96; ASYS Hitech, Cambridge, UK) and the provided software (Kim, ver.5.45.0.1, Daniel Kittrich, Prague, Czech Republic).

Proteins extracted from muscle samples were analyzed by western blotting as previously described (Cumming et al. 2014). Briefly, equal amounts of protein were loaded per well (7–28 *μ*g) and separated in 4–12% SDS‐PAGE gels (Cat#NP0321, NuPAGE; Invitrogen, Life technologies) under denatured conditions. Proteins were transferred to PVDF‐membranes (Cat#162‐0177; Bio‐Rad), before being blocked in a 5% fat‐free skimmed milk and 0.05% TBS‐t solution (TBS, Cat#170‐6435, Bio‐Rad; Tween 20, Cat#437082Q, VWR International, Radnor, PA; Skim milk, Cat#1.15363; Merck, Darmstadt, Germany). Antibodies against GPx1 (rabbit‐anti GPx1, Cat#ab22604; Abcam, Cambridge, UK), mnSOD (mouse‐anti mnSOD, Cat#ab16956; Abcam), NF*κ*B p65 (anti‐rabbit NF*κ*B p65, Cat#ab7970; Abcam), I*κ*B*α* (anti‐rabbit I*κ*B*α*, Cat#ab32518; Abcam), HSP70 (mouse‐anti HSP70, Cat#ADI‐SPA‐810; Enzo Life Sciences, Farmingdale, NY) or *α*B‐crystallin (mouse‐anti *α*B‐crystallin, Cat#ADI‐SPA‐222; Enzo Life Sciences), and appropriate secondary antibodies (goat anti‐mouse, Cat#31430; Thermo Scientific, Rockford, IL, or Goat anti‐rabbit, Cat#7074; Cell Signaling Technology, Danvers, MA) were used. Bands were visualized using a HRP‐detection system (Super Signal West Dura Extended Duration Substrate, Cat#34076; Thermo Scientific). Chemiluminescence was measured using a CCD image sensor (Image Station 2000R or Image Station 4000R; Eastman Kodak Inc., Rochester, NY) and band intensities were calculated with the Carestream molecular imaging software (Carestream Health Inc., Rochester, NY). All samples were run as duplicates and mean values were used for statistical analyses.

GPx1, I*κ*B*α*, HSP70 was analyzed using the cytosolic fraction, mnSOD using the membrane‐ and cytosolic fraction (results represents the combined results from both fractions), *α*B‐crystallin using the cytosolic and cytoskeletal fraction (only cytosolic fraction for the pre‐post results) and NF*κ*B p65 using the cytosolic‐ and nuclear fraction.

HSP27 was measured in the cytosolic and cytoskeletal fractions with an in house‐made double antibody sandwich ELISA using a monoclonal capture antibody against HSP27 as previously described (Cumming et al. 2014).

#### RT‐qPCR

Total RNA was extracted from muscle biopsies (*n* = 10) from the acute study by homogenization of samples in TRIzol reagent (Cat# 15596; Invitrogen, Life Technologies) according to the manufacturer's procedures. DNase I digestion was performed using RNase free‐DNase from Qiagen (Cat#79254; Qiagen Inc., Germantown, MD) in order to prevent genomic DNA contamination. Quantitative‐PCR (q‐PCR) analysis was performed in an Applied Biosystems 7500 Real‐Time PCR System (Applied Biosystems, Foster City, CA, USA). qPCR reactions were performed using Power SYBR Green RNA‐to‐Ct ^™^ 1‐step Kit (Cat# 4389986; Applied Biosystems) supplemented with forward and reverse primer in a total volume of 20 *μ*L. The reverse transcription step was performed co‐incubating the PCR primers with 6 ng of RNA and the MultiScribe^™^ Reverse Transcriptase (Cat#4311235; Invitrogen, Life Technologies) at 48°C for 30 min in the presence of RNase Inhibitor. Thermocycling conditions were according to the recommendations of the manufacturer. *Ct* values for gene expression were calculated according to the comparative *Ct* method (Pfaffl 2001). Relative quantification was performed by simultaneous quantification of *GAPDH* and *18S* gene expression. The primers used for RT–qPCR analyses are listed in Table 1.

**Table 1.** Human primer sequences used for RT‐qPCR

Gene name (accession no.)	Sense	Antisense
*CRYAB* (NM_001885)	GTCAACCTGGATGTGAAGCA	TTTTCCATGCACCTCAATCA
*HSPA4* (NM_002154)	TTAAGTCCAAAATCCGTGCAT	CTGAAGCATTTGCACTCATCA
*S0D2* (NM_000636)	CCCTGGAACCTCACATCAAC	GGTGACGTTCAGGTTGTTCA
*GPx1* (NM_000581)	ACGATGTTGCCTGGAACTTT	TCGATGTCAATGGTCTGGAA

#### Muscle glutathione (GSH)

Muscle GSH was analyzed by a commercial company (Vitas AS, Oslo, Norway) using a reagent kit originally intended for measurement of total homocysteine in plasma by HPLC (Cat#195‐4075, Bio‐Rad). The kit was modified and validated for quantification of total glutathione in tissues, with a detection limit (LOQ) of 0.04 mmol/L. All samples were analyzed in triplicate (CV% <2%).

### Blood sampling and analysis

Venous blood was drawn in the morning after overnight fasting. Heparin and EDTA coated tubes were centrifuged at 1500 *g* for 10 min at 4°C. Heparin plasma destined for vitamin C analysis was mixed in equal volumes with metaphosphoric acid before freezing; the further analysis procedure is described by Karlsen et al. (2005). Vitamin E was analyzed in EDTA plasma, as described by Bastani et al. (2012). Plasma glutathione analyses (GSH and GSSG) followed procedures as described by Sakhi et al. (2006). Uric acid and total antioxidant status (TAS) in plasma was measured by a colormetric enzymatic method on an automatic analyzer (MaxMat PL, MaxMat S.A., Montpellier, France). Detection of uric acid was achieved by reagents from MaxMat S.A. (Cat#RM URAC0200V). To ensure adequate quality, multiparametric control sera, two levels of controls, one close to the normal patient values (Maxtrol N, Cat#RM MNCO0030V) and a second representative of pathologic values (Maxtrol P, Cat#RM MPCO0030V) from MaxMat S.A. was used. TAS was measured by incubating the sample with a peroxidase (metmyoglobin) and H_2_O_2_ to produce the radical cation ABTS (2,2′‐Azino‐di‐[3‐ethylbenzthiazoline sulphonatel]). Quantification of TAS was achieved by reagents and standards (Cat#NX 2332) and control (Cat#NX 2331) from Randox Laboratories (Randox Laboratories Ltd., Crumlin, UK).

### Statistics

All values are presented as means ± standard deviations (SD). All data were tested for normality with a Shapiro–Wilk normality test. If data were not normally distributed, log‐transformation was applied to achieve normality before further analyses. A two‐way ANOVA was used to evaluate the effects of training (pre‐post‐training intervention and acute exercise session) and supplementation, and a Holm–Sidak multiple comparisons test was applied for post hoc analyses. Unpaired *t*‐test was used to evaluate differences between groups in participant characteristics and variables for exercise intensity (Percentage of HF_max_ and VO_2max_, and lactate) in the acute experiment. Correlations were tested using the Pearson product‐moment correlation coefficient test. Effect sizes for mRNA data was calculated and reported using Cohen's *d*. An effect size of 0.2 was considered small, 0.5 medium, 0.8 large, and 1.3 very large. Figures display max–min values, 25th and 75th quartiles and medians (boxplot). Outliers defined by Tukey's rule are shown in figures. The level of significance was set to *P *<**0.05, and *P* values are reported as multiplicity‐adjusted *P* values. Trends were defined as *P *=**0.05–0.1. Graphpad Prism 6 (GraphPad Software Inc., La Jolla, CA) was used for statistical analyses.

## Results

The two groups were not different at baseline (pre‐training) regarding training experience, VO_2max_, age, body weight or height (Table 2). Furthermore, the groups completed the same number of training sessions, exercised with equal training intensity (heart rate monitoring and perceived exertion), and ingested similar levels of energy (data not shown; see ref. Paulsen et al. 2009).

**Table 2.** Baseline characteristics of the participants included in the study

Variable	Group	
	Vitamin C and E	Placebo
Age (years)		
All	24 ± 4	23 ± 3
Males	25 ± 5	24 ± 4
Females	24 ± 4	22 ± 1
Body weight (kg)		
All	71 ± 13	70 ± 13
Males	79 ± 13	80 ± 10
Females	63 ± 8	60 ± 6
Height (cm)		
All	176 ± 11	176 ± 10
Males	184 ± 10	183 ± 5
Females	168 ± 4	168 ± 6
Training hours per week		
All	3.8 ± 1.8	5.4 ± 5.8
Males	3.6 ± 1.5	6.2 ± 6.9
Females	3.9 ± 2.2	4.2 ± 3.8
VO_2max_ (mL/kg/min)		
All	54.6 ± 8.6	54.2 ± 7.4
Males	59.9 ± 8.4	58.0 ± 6.6
Females	50.1 ± 5.7	49.8 ± 6.0

Values are mean ± standard deviations.

### Vitamin C and E concentration in plasma

**Figure 1.**
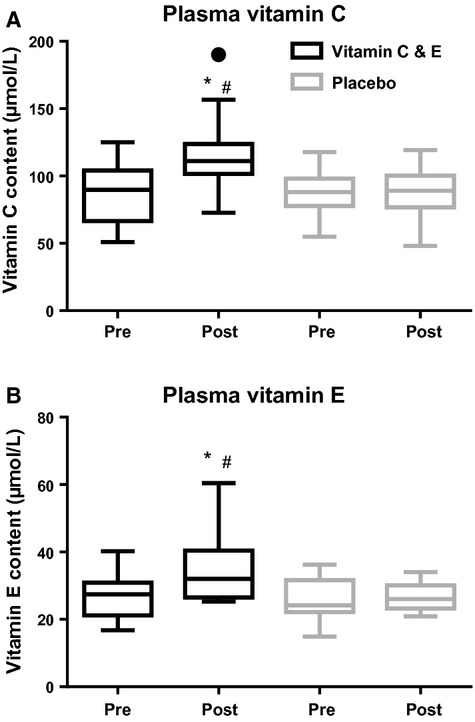
Plasma vitamin C content (ascorbic acid + dehydroascorbic acid) (A) and plasma vitamin E content (B) for the vitamin C and E‐ (black) and placebo group (gray) before and after 11 weeks of training. ●:Outlier (Tukey's rule); #:difference between groups (*P *<**0.05)*;* *:different from pre (*P *<**0.05).

At baseline, the groups did not differ in plasma vitamin C or E concentration. The vitamin C and E group increased basal plasma vitamin C and E concentration after 11 weeks of supplementation (*P *=**0.004), with higher concentrations compared to the placebo group (43 ± 53 vs. 3 ± 32%, and 31 ± 27 vs. 8 ± 28%, for vitamin C and E, respectively; *P *<**0.001; Fig. 1).

### Acute response to supplementation and exercise

**Figure 2.**
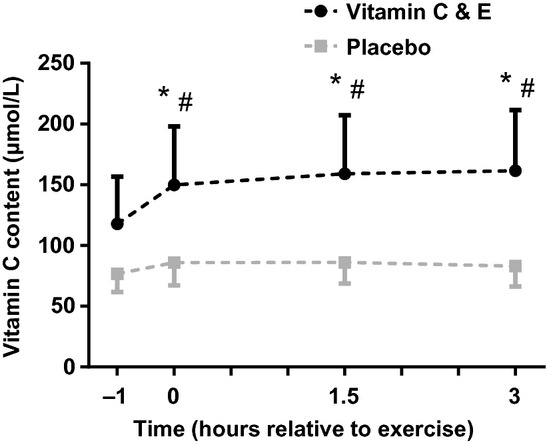
Plasma vitamin C (ascorbic acid + dehydroascorbic acid) content for the vitamin C and E‐ (black) and placebo group (gray) after ingestion and the acute exercise bout exercise (at time point 0); #:difference between groups (*P *<**0.05)*;* *:different from pre (time point −1; *P *<**0.05).

The vitamin C and E supplementation increased plasma vitamin C concentration the first hour after ingestion (Fig. 2), and it remained elevated for 3 h after ingestion (*P *=**0.047). The placebo group did not show any significant changes in plasma vitamin C concentration during or after the exercise session.

The exercise intensity during the acute exercise experiment was not different between groups (Table 3): Mean of all intervals; percentage of HR_max_; 93 ± 1 versus 92 ± 2%, percentage of VO_2max_; 89 ± 3 versus 88 ± 3%, blood lactate; 4.2 ± 1.1 versus 3.9 ± 0.9 mmol/L for the vitamin C and E and placebo group, respectively.

**Table 3.** Intensity variables during the acute exercise bout

Variable	Interval 1	Interval 2	Interval 3	Interval 4
% HF_max_				
Vitamin C and E	90 ± 1	92 ± 1	94 ± 1	95 ± 1
Placebo	90 ± 2	91 ± 2	92 ± 1	93 ± 1
% VO_2max_				
Vitamin C and E	86 ± 3	88 ± 3	90 ± 4	92 ± 5
Placebo	87 ± 4	88 ± 4	89 ± 3	89 ± 5
La^−^ (mmol/L)				
Vitamin C and E	3.3 ± 1.0	3.9 ± 0.8	4.4 ± 1.3	5.2 ± 1.7
Placebo	3.1 ± 0.9	3.9 ± 0.9	4.2 ± 1.2	4.3 ± 1.2

Values are mean ± standard deviations.

**Figure 3.**
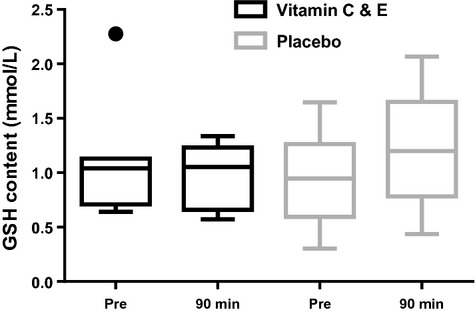
Muscle GSH content for the vitamin C and E‐ (black) and placebo group (gray) pre and 90 min after exercise. ●:Outlier (Tukey's rule).

Ninety minutes after exercise, no changes were observed in skeletal muscle glutathione (GSH) content (Fig. 3).

**Figure 4.**
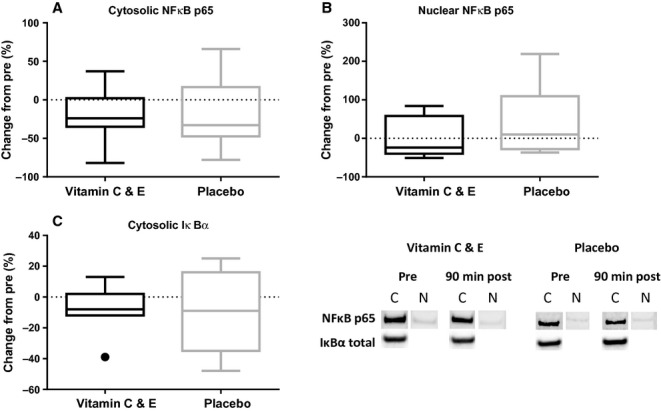
Relative change in muscle cytosolic NF*κ*B p65 (A), nuclear NF*κ*B p65 (B) and total cytosolic I*κ*B (C) measured by protein immunoblot for the vitamin C and E‐ (black) and placebo group (gray) 90 min after exercise. ●:Outlier (Tukey's rule). C, cytosolic fraction; N, nuclear fraction.

A tendency (*P *=**0.082) toward decreased cytosolic NF*κ*B p65 content was observed 90 min after exercise, with no effect of supplementation (Fig. 4A). No significant changes were observed 90 min after exercise in nuclear NF*κ*B p65 (Fig. 4B) and cytosolic I*κ*B*α* content (Fig. 4C).

**Figure 5.**
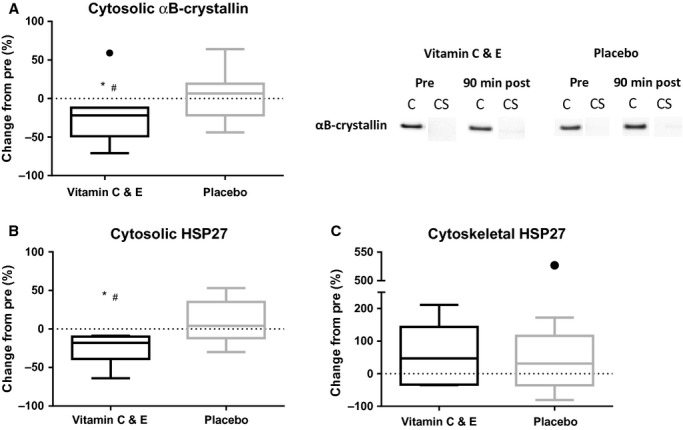
Relative change in muscle cytosolic *α*B‐crystallin (A), measured by protein immunoblot, and cytosolic HSP27 (B) and cytoskeletal HSP27 (C) measured by ELISA for the vitamin C and E‐ (black) and placebo group (gray) acutely after exercise. ●:Outlier (Tukey's rule); #:difference between groups (*P *<**0.05)*;* *: different from pre (*P *<**0.05). C, cytosolic fraction; CS, cytoskeletal fraction fraction.

Ninety minutes after exercise, the vitamin C and E group showed decreased *α*B‐crystallin content in the cytosolic fraction compared to pre values (*P *=**0.021) and placebo (*P *=**0.002; Fig. 5A). *α*B‐crystallin in the cytoskeletal fraction showed low or non‐detectable levels independently of group (data not shown). The vitamin C and E group showed decreased HSP27 content in the cytosolic fraction 90 min after the interval bout compared to pre‐values (*P *=**0.030; Fig. 5B) and the placebo group (−26 ± 20 vs. 9 ± 29%, respectively; *P *=**0.014). A slight tendency (*P *=**0.117; Fig. 5C) to increased HSP27 amount was observed in the cytoskeletal fraction 90 min after exercise in both groups, with no effects of supplementation.

**Figure 6.**
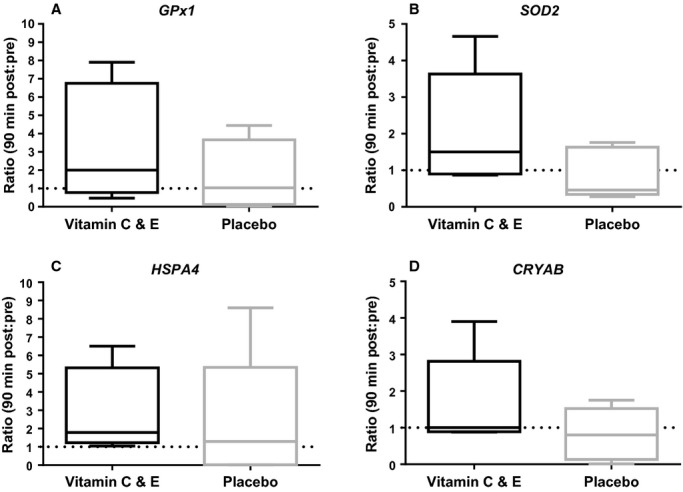
Muscle mRNA expression‐ratio (Post:Pre) for *GPx1* (A), *SOD2* (B), *HSPA4* (C) and *CRYAB* (D) measured by RT‐qPCR for the vitamin C and E‐ (black) and placebo group (gray) 90 min after exercise (*n* = 10).

Except for a tendency to increased *GPx1* mRNA expression 90 min postexercise (*P *=**0.096; Fig. 6A), *SOD2* (mnSOD), *HSPA*4 (HSP70) or *CRYAB (α*B‐crystallin) mRNA expression did not change significantly relative to pre‐exercise values (Fig. 6B–D). Although the expression of the investigated genes was not significantly affected by the vitamin C and E supplementation, effect size calculations revealed effect sizes >0.6 between groups; *GPx1 ES *= 0.64 (medium), *SOD2 ES *= 1.01 (large), *CRYAB ES *= 0.82 (large), *HSPA4 ES *= 0.64 (medium).

### Chronic effects of training and supplementation on skeletal muscle variables

**Figure 7.**
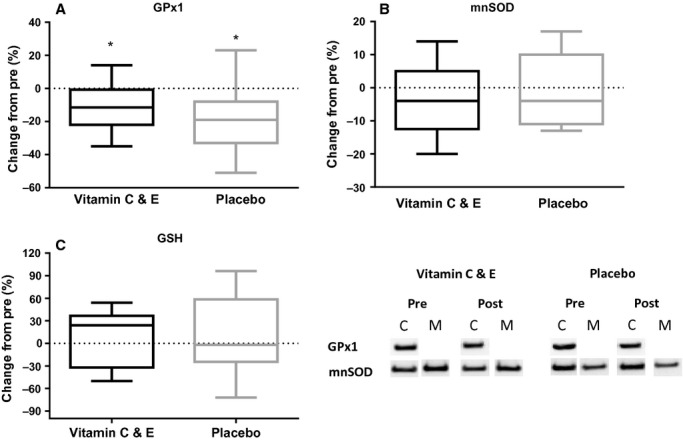
Changes in protein levels for GPx1 (A), mnSOD (B) and GSH (C) measured by protein immunoblot for the vitamin C and E‐ (black) and placebo group (gray) after 11 weeks of training. *:different from pre (*P *<**0.05). C, cytosolic fraction; M, membrane fraction.

**Figure 8.**
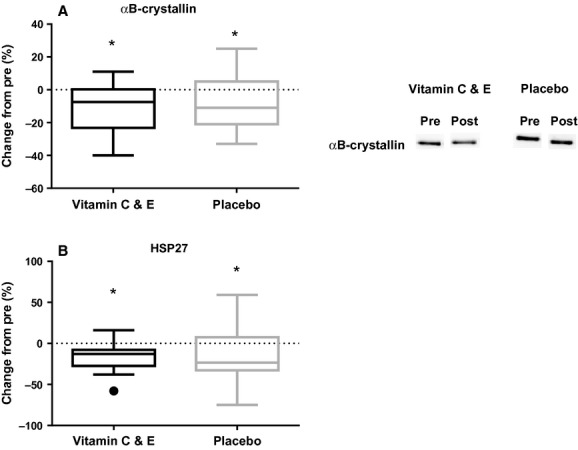
Changes in protein levels for *α*B‐crystallin (A), measured by protein immunoblot, and HSP27 (B) measured by ELISA for the vitamin C and E‐ (black) and placebo group (gray) after 11 weeks of training. ●:Outlier (Tukey's rule); *:different from pre (*P *<**0.05).

After 11 weeks of endurance training, muscle GPx1 levels were decreased by −11 ± 14% in the vitamin C and E group and by −19 ± 18% in the placebo group (*P *<**0.001; Fig. 7A), with no effects of supplementation. No changes were observed for muscle mnSOD levels (Fig. 7B), muscle GSH levels (Fig. 7C), or cytosolic HSP70 levels (Paulsen et al. 2014). The *α*B‐crystallin levels were decreased by −10 ± 14% in the vitamin C and E group and −9 ± 17% in the placebo group (*P *<**0.001; Fig. 8A), with no effects of supplementation. After 5–6 weeks of training (at the commencement of the acute exercise‐induced stress test), the vitamin C and E group had unchanged HSP27 levels compared to pre‐values (6 ± 17%), while the placebo group displayed a 24 ± 32% reduction (*P *=**0.016). In the end of 11 weeks of training, HSP27 amount was decreased by −17 ± 17% in the vitamin C and E group and −14 ± 33% in the placebo group (*P *<**0.002; Fig. 8B), with no effects of supplementation.

### Chronic effects of training and supplementation on plasma variables

A significant decrease in uric acid was observed in the vitamin C and E group by −24 ± 21% compared to the placebo group (6 ± 27%; *P *<**0.001; Table 4) after training. Plasma GSH increased after 11 weeks of training in both groups (*P *=**0.003; Table 4), with no effect of supplementation. Oxidized glutathione (GSSG) did not change after training, whereas total GSH (GSH + GSSG) increased after training in both groups (*P *=**0.003; Table 4), with no effect of supplementation. The ratio between GSH:GSSG did not change after training (Table 4). Total antioxidant capacity (TAS) did not change after training or supplementation (Table 4).

**Table 4.** Changes in plasma variables before and after 11 weeks of endurance training

Variable	Pre‐training	Post‐training	%‐Change	Level of significance		
				Group	Training	Interaction
GSH (*μ*mol/L)						
Vitamin C and E	2.4 ± 0.5	2.8 ± 0.7	23 ± 38	*P* = 0.991	*P* = 0.003	*P* = 0.577
Placebo	2.3 ± 0.7	2.9 ± 1.1	32 ± 53			
GSSG (*μ*mol/L)						
Vitamin C and E	0.08 ± 0.05	0.1 ± 0.08	50 ± 101	*P* = 0.479	*P* = 0.362	*P* = 0.223
Placebo	0.08 ± 0.05	0.08 ± 0.04	18 ± 77			
Total GSH (*μ*mol/L)						
Vitamin C and E	2.5 ± 0.6	2.95 ± 0.6	23 ± 37	*P* = 0.950	*P* = 0.003	*P* = 0.637
Placebo	2.4 ± 0.7	3.0 ± 1.1	31 ± 51			
GSH:GSSG						
Vitamin C and E	36.2 ± 18.5	41.4 ± 28	38 ± 109	*P* = 0.659	*P* = 0.136	*P* = 0.592
Placebo	35.8 ± 16	46.8 ± 29.8	59 ± 115			
Uric acid (*μ*mol/L)						
Vitamin C and E	191.5 ± 53.6	146.1 ± 50.6	−24 ± 21	*P* = 0.06	*P* = 0.021	*P* < 0.001
Placebo	200.3 ± 61.7	210.8 ± 77.8	6 ± 27			
TAS (mmol/L)						
Vitamin C and E	1.40 ± 0.10	1.38 ± 0.08	−2 ± 4	*P* = 0.101	*P* = 0.830	*P* = 0.467
Placebo	1.45 ± 0.10	1.46 ± 0.07	1 ± 6			

GSH, reduced glutathione; GSSG, oxidized glutathione; Total GSH, GSH + GSSG; TAS, total antioxidant status.

Values are mean ± standard deviations.

### Correlations

The changes observed in mRNA expression acutely after the standardized exercise bout performed in the middle of the training period did not correlate with changes in protein levels during the entire training period for either GPx1, mnSOD, HSP70 or *α*B‐crystallin.

## Discussion

The effects of antioxidants on the adaptations to exercise have been studied by a number of research groups, often with conflicting results. By means of a double blind randomized placebo‐controlled trial we investigated the effects of vitamin C and E supplementation on the acute stress response to exercise and training adaptations in the antioxidant systems and heat shock proteins after 11 weeks of endurance training. The main findings were that the acute reductions in cytosolic *α*B‐crystallin and HSP27 content, as well as the stress‐related gene expression response, were more pronounced in the vitamin C and E group than in the placebo group 90 min after exercise. Furthermore, we observed that GPx1, *α*B‐crystallin, and HSP27 decreased after 11 weeks of endurance training, but antioxidant supplementation did not have any beneficial or detrimental effects on these variables. Vitamin C and E supplementation did, however, markedly decrease uric acid content in plasma. Finally, endurance training per se increased GSH content in plasma.

### Acute response to exercise

The intensity during the interval session was high and similar for both groups, as indicated by the heart rate, VO_2_, and blood lactate levels. Despite the high exercise intensity, no detectable degradation of I*κ*B*α* or translocation of NF*κ*B p65 to the nuclei was observed 90 min after exercise. Consequently, the stress induced during the exercise session appeared insufficient in activating the NF*κ*B pathway. Alternatively, the timing of the biopsy (90 min after exercise) might not have been optimal for detecting NF*κ*B activation, since NF*κ*B p65 in the nucleus has been shown to peak 2 h after exhaustive running in rats (Hollander et al. 2001).

We observed no significant increase in antioxidants (*GPx1* and *SOD2*) or HSP (*CRYAB* and *HSPA4*) mRNA expression after the exercise session. However, there might be a possibility that we missed the alterations of these mRNAs, which could occur at a later time point, and the low number of participants included in these analyses also reduced the statistical power. Interestingly, effect sizes indicate that the vitamin C and E group, in general, had higher mRNA expression of *GPx1*,* SOD2*,* CRYAB*, and *HSPA4*, relative to the placebo group after the interval session. In line with these observations, Yfanti et al. (2012) reported overall higher basal GPx1 mRNA expression in the antioxidant supplemented group.

A larger stress response to the interval session in the vitamin C and E group was further supported by the significant reductions in soluble *α*B‐crystallin and HSP27 observed 90 min after exercise. The decreased *α*B‐crystallin and HSP27 levels in the cytosolic fraction indicate a rapid translocation to stressed cell structures, as seen in experiments with eccentric exercise (Koh and Escobedo 2004; Paulsen et al. 2009).

Seemingly, the increased stress response observed in the vitamin C and E group is in contrast to our initial hypothesis of decreased exercise‐induced stress by antioxidant supplementation. However, the interval session was conducted after 5–6 weeks of combined supplementation and training, and we suggest that the placebo group tolerated the stress from the intervals better than the vitamin C and E group because of more adequate training adaptations in this period. More precisely, mitochondrial adaptations to the endurance training was attenuated in the vitamin C and E group (Paulsen et al. 2014), and this might have caused higher exercise‐induced stress in the vitamin C and E group than in the placebo group. Furthermore, decreased muscle HSP27 levels during the first 5–6 weeks of training only observed in the placebo group supports a more rapid and adequate training adaptation in the placebo group compared to the vitamin C and E group (discussed later).

### Chronic adaptations to training and supplementation on skeletal muscle variables

#### Antioxidant systems

No observable increases in mnSOD levels during 11 weeks of training was surprising, as endurance training has been shown to increase mnSOD in animals (Higashida et al. 2011) and trained humans (Morton et al. 2008; Yfanti et al. 2010). Lack of increases in mnSOD might, however, be explained by initially high levels in our well‐trained participants. As a consequence of no changes in mnSOD levels with training, it could not be expected to find any blunting effect of vitamin C and E supplementation. A possible interference from supplementation could, therefore, be hidden by initial high protein levels, and it might be a chance that untrained individuals would experience different outcomes than in this study.

Contrary to mnSOD, GPx1 was decreased in both groups after training. These findings are, in agreement with Gliemann et al. (2013), who reported that 8 weeks of high‐intensity endurance training decreased GPx1 protein content in initially inactive elderly men, and with no effects of resveratrol supplementation.

As recently published, vitamin C and E supplementation blunted the endurance training induced increase in mitochondrial content (as measured by COX4 protein content) in the same participants as in the present study (Paulsen et al. 2014). The fact that we did not observe any group differences in mnSOD (and GPx1) content in the present study, indicates that oxidative enzymes in the mitochondria and mnSOD are differently regulated. We suggest that the increases in mitochondrial content in the placebo group, with no increases in mnSOD, indicate a better mitochondrial function with less electron leakages during exercise. The improved mitochondrial function might in addition reduce hydrogen peroxide (H_2_O_2_) in cytosol (Venditti et al. 2014); which might contribute to the observed reduction in GPx1 levels. In the vitamin C and E group, the antioxidant supplementation hampered the mitochondrial adaptations, but in contrast to the placebo group, the antioxidant function of the supplements induced more substrate for RONS scavenging. This can, at least in theory, explain decreased GPx1 content in the vitamin C and E group. Unchanged GSH levels measured in muscle homogenate could be explained by the fact that increases in GSH have been seen in muscles containing mostly type 2 fibers (Sen et al. 1992; Leeuwenburgh et al. 1997). As our participants had 51% type 2 fibers in the investigated muscle (Paulsen et al. 2014), any increases may have been camouflaged by potential fiber‐specific changes.

#### Heat shock proteins

The acute stress response to the high‐intensity interval session observed in the vitamin C and E group normally results in increased expression of heat shock proteins (Morton et al. 2009b). This was, however, not reflected in the biopsies taken after the training period as *α*B‐crystallin and HSP27 levels were decreased in both groups. Cytosolic HSP70 protein levels were unchanged after the training period, but two participants (one in each group) displayed a very large increase in HSP70 after training (could be defined as outliers). When removing these values from the data set, a significant decrease (*P *<**0.001) was observed in HSP70 levels as well.

Decreased HSP levels during the training period was unexpected, because previous studies have found that endurance training increases HSP levels in skeletal muscles (Yoshioka et al. 2003; Morton et al. 2008; Morton et al. 2009a). Interestingly, we observed that the vitamin C and E group had unchanged HSP27 levels 5–6 weeks into the training period (data from the acute exercise bout) compared to pre‐values, whereas the placebo group had decreased HSP27 levels at this time point. Since the HSPs play an important role in cell stress homeostasis, these findings support our hypothesis that more exercise stress was generated in the vitamin C and E group during the initial 5–6 weeks of training. Nevertheless, because the HSP27 levels were decreased similarly in both groups after 11 weeks of endurance training, it seems like this effect was transient and related to the first weeks of supplementation.

### Chronic adaptations of training and supplementation on plasma variables

Due to high plasma concentration and antioxidant function of uric acid (Ames et al. 1981; Glantzounis et al. 2005), plasma total antioxidant status (TAS) measurements are highly dependent on the uric acid concentration. Although, plasma uric acid levels decreased in the vitamin C and E group after training, TAS did not. This was probably because of the increased plasma vitamin C levels, thus, the overall antioxidant capacity in plasma was held stable.

In contrast to muscle GSH, plasma GSH and total plasma GSH levels (GSH + GSSG) increased significantly as an effect of training. This is in agreement with a previous cross‐sectional study reporting higher levels of plasma GSH in trained compared to untrained individuals (Kretzschmar et al. 1991). There is a lack of studies investigating plasma GSH after endurance training, but a few animal studies report no changes in response to training (Leeuwenburgh et al. 1997; Ohkuwa et al. 1997). Despite being trained before entering the study, the participants in the present study increased plasma GSH and total plasma GSH over the training period. This indicates that the training stress was sufficient to induce upregulation of plasma GSH levels even in trained subjects. The vitamin C and E supplementation induced no additional effects on plasma GSH. However, vitamin C supplementation alone has been demonstrated to increased GSH in red blood cells (Johnston et al. 1993) and lymphocytes (Lenton et al. 2003). Thus, we cannot exclude a possible effect on other GSH fractions.

## Conclusion

The present study investigated the effects of vitamin C and E supplementation on acute responses to exercise and long‐term (11 weeks) adaptations to endurance training on antioxidant systems and heat shock proteins. The results show that vitamin C and E supplementation did not significantly affect the acute activation of the NF*κ*B pathway. However, the acute reductions observed in cytosolic *α*B‐crystallin and HSP27 together with the more pronounced expression of stress genes after the standardized interval session indicate that the exercise‐induced stress measured in the middle of the intervention was less tolerated in the vitamin C and E group. The training decreased muscle GPx1, *α*B‐crystallin and HSP27 levels, whereas no changes were observed in mnSOD and muscle GSH levels. Plasma GSH increased in both groups. Vitamin C and E supplementation decreased plasma uric acid levels, but plasma total antioxidant status was stable, likely because of the concomitant increase in plasma vitamin C and E levels. We conclude that vitamin C and E supplementation did not negatively affect the training‐induced adaptations in muscle antioxidant systems or heat shock proteins in healthy previously trained participants. However, indications of less tolerability toward exercise‐induced stress in the vitamin C and E group could in the long‐term, or during periods of very intense training, delay recovery, and not cause optimal gains in physical performance.

## Conflict of Interest

None declared.
